# Significance of radiology in the diagnosis and management of ruptured left gastric artery aneurysm associated with acute pancreatitis: Case report: Erratum

**DOI:** 10.1097/MD.0000000000015121

**Published:** 2019-03-15

In the article, “Significance of radiology in the diagnosis and management of ruptured left gastric artery aneurysm associated with acute pancreatitis: Case report”,^[1]^ which appeared in Volume 98, Issue 10 of *Medicine*, all instances of the abbreviation GGA should be GAA.
